# *Codium fragile* (Suringar) Hariot as Biostimulant Agent to Alleviate Salt Stress in Durum Wheat: Preliminary Results from Germination Trials

**DOI:** 10.3390/plants13020283

**Published:** 2024-01-18

**Authors:** Angelo Rossini, Roberto Ruggeri, Nada Mzid, Francesco Rossini, Giuseppe Di Miceli

**Affiliations:** 1 Department of Agriculture and Forest Sciences, University of Tuscia, Via S. Camillo de Lellis, 01100 Viterbo, Italy; angelo.rossini@unitus.it (A.R.); r.ruggeri@unitus.it (R.R.); nada.mzid@unitus.it (N.M.); 2 Department of Agricultural, Food and Forestry Sciences (SAAF), University of Palermo, Viale delle Scienze, Ed. 5, 90128 Palermo, Italy; giuseppe.dimiceli@unipa.it

**Keywords:** *Codium fragile* (Suringar) Hariot, durum wheat, salt stress, seaweed extract, *Triticum turgidum* L. subsp. *durum* (Desf.) Husn

## Abstract

Soil salinization is a critical environmental problem in arid and semiarid regions of the world. The aim of the present study was to evaluate the effect of an algae-based biostimulant on germination and seedling vigour of durum wheat (*Triticum turgidum* L. subsp. *durum* (Desf.) Husn.), under different saline conditions (0, 100, and 200 mM NaCl). The experiment was carried out under controlled-environment conditions. Seeds were sprayed with a solution containing a combination of fungicide and different concentrations of *Codium fragile* (Suringar) Hariot algae (0%*_w_*_/*v*_, 10%*_w_*_/*v*_, 20%*_w_*_/*v*_, and 30%*_w_*_/*v*_). All experimental units were placed in a germination cabinet. The effect of the seaweed extract (SWE) on seed germination and seedling performance under salinity stress was evaluated over a period of 8 days. Coleoptile length and biomass were found to be significantly and positively affected by the application of different SWE doses as compared to the control treatment (0% algae). As for germination traits, seeds treated with SWE showed a final germination (from 82% to 88%), under severe saline conditions, significantly higher than that observed in the control treatment (61%). Our findings indicate that the appropriate dose of biostimulant can markedly improve the germination and the seedlings vigour of durum wheat seeds under saline conditions. Additional studies will be needed to understand the mechanism of action of this biostimulant and its effectiveness in longer studies under field conditions.

## 1. Introduction

Soil salinization is one of the major factors contributing to the loss of agricultural productivity [1]. Every year, around 10 million hectares of world agricultural land are degraded by salinity [2]. In Europe, salt-affected soils cover an area of about 31 million ha, particularly concentrated in the Mediterranean Basin [3,4,5]. Salinization due to seawater intrusion, or due to irrigation with poor quality water, is cause of serious concern in rural areas of Cyprus, Greece, Italy, Portugal, and Spain [4,6]. In the European Union alone, 1 million hectares are affected by soil salinity [7]. It was estimated that 25% of irrigated Mediterranean cropland is prone to moderate or high salinization [8], and by 2050, 50% of the world’s arable land will be subjected to salinity [9]. High concentrations of salts have detrimental effects on crop development, especially in the phases of seed germination and seedling growth [10,11,12].

Among cereals, wheat represents the staple crop for 40% of the world’s population, with a current annual production of 790.6 million tons [13], particularly for people living in Europe, North America, the Western and Northern parts of Asia, and the Americas [14]. Although durum wheat (*Triticum turgidum* L. subsp. *durum* (Desf.) Husn.) accounts for a small proportion of the global wheat production (around 7%), it has ever been a crucial commodity for countries surrounding the Mediterranean Sea [15]. Durum wheat was used as the model plant because of its importance as a crop in arid and semiarid areas of the Mediterranean Basin [16], and because is one of the most salt-sensitive cereal crops, especially during seed germination [17,18,19,20,21]. A high salinity level can result in 50% grain yield reduction [22]. Salt tolerance during germination and seedling establishment is fundamental for the future development and growth of crops, particularly in arid and semi-arid areas [20,23]. For that reason, studies aiming to find a sustainable way to guarantee a satisfying yield in moderately saline soils are not only warranted but also strongly sought after [24,25].

World population growth and climate change are severely threatening the global food security and environmental sustainability [26,27]. Therefore, the ambitious target of the primary sector for the future (and present) is to produce more food while impacting less on natural resources. In agriculture, efforts are being made in different directions including the reduction of chemical fertilizers’ use [28,29,30]. In this context, biostimulants are receiving a lot of attention from both researchers and farmers as natural substances able to enhance crop performances [31,32,33]. The use of biostimulants to alleviate the effects of abiotic stressors such as salinity is a promising frontier for crop management.

Biostimulants, especially seaweed extracts (SWE), showed good results in promoting seed germination and seedlings vigour of different plant species under both optimal and saline conditions [34,35,36,37,38,39,40,41,42,43,44]. The positive effect is probably due to the high content of phytohormones, which tend to play a major role in seed germination [45]. However, few studies have been conducted until now on the effect of SWE on seed germination of durum wheat under saline conditions. Moreover, indications on the optimal dose of SWE-based biostimulants for seed treatments are scarce in the scientific literature. Hydropriming proves to be the method most used to treat seeds in experimental trials. However, this technique, consisting of soaking seed for a period in a solution containing a certain amount of SWE [46], does not represent a viable method for large-scale applications in seed industry because primed seeds are extremely difficult to manage [47]. Therefore, methods of delivering biostimulants as seed coatings, possibly together with other active ingredients, need to be explored to speed the technology transfer [48].

Among algae species from which SWE are obtained, *Codium fragile* (Suringar) Hariot, a green seaweed that lives in some salt marshes located in Tunisia, is one of the most invasive of the Mediterranean coasts [49,50]. Some studies showed promising results derived from the application of *Codium* spp. extract to enhance seed germination and seedlings performance of different crop species [46,51,52,53].

The objective of this study was to assess the effect of different concentrations of *Codium fragile* extract (0%*_w_*_/*v*_, 10%*_w_*_/*v*_, 20%*_w_*_/*v*_, and 30%*_w_*_/*v*_) delivered as a seed coating on germination and early development of durum wheat seeds, under optimal (0 mM NaCl) and rising saline conditions (100 and 200 mM NaCl). In particular, the following hypotheses were tested: (i) seed treatment with SWE improves germination features and seedling vigour as compared to control and, above all, under salt stress conditions; (ii) the effect is proportional to the applied dose.

To verify these hypotheses, an experiment under controlled environment conditions was set up, randomizing the different treatments (salinity levels and SWE application doses) within the germination cabinet and evaluating germination and seedling traits at the completion of the trial.

## 2. Materials and Methods

### 2.1. Algal Collection and Extract Preparation

*Codium fragile* was collected in the salt marshes of South-East Tunisia. Samples were washed several times, first with fresh water and then with distilled water, air-dried, cut into pieces, and finally powdered with a mixed grinder. Ten grams of the obtained powder were suspended with 1 L of distilled water under continuous stirring at 300 rpm with a heating magnetic stirrer for 2 h at 50 °C [54]. Subsequently, the sample was put into an ultrasonic extractor for 45 min, then filtered using Whatman No. 1 filter paper to obtain 100% algal extract and stored at 4 °C for further analysis and treatments [55].

### 2.2. Experimental Design

Three different artificial growth conditions were used during the experiment:

- OPTIMAL: 0 mM of NaCl, control treatment;
- MID-SALINE: 100 mM of NaCl, a salinity level at which wheat begins to experience distress [56];
- HIGH-SALINE: 200 mM of NaCl, a salinity level considered an extreme condition [57].

Durum wheat seeds (cultivar Monastir) were selected for apparent uniformity in size and shape and treated with a solution containing the following ingredients:

- A liquid fungicide, usually used for cereals coating in Europe, at a dose of 200 g for 100 kg of seed, containing 2.34% Fludioxonil, 2.34% Difenoconazole, and 0.93% Tebuconazole;
- *Codium fragile* extract at 10 g dry matter (g_dm_) L^−1^;
- Distilled water.

Monastir was chosen because it is a modern durum wheat cultivar extensively cultivated around the Mediterranean basin [58] and it is reported to be a cultivar with medium susceptibility to salinity stress [24].

Four seed dressing solutions were prepared, changing just the amount of *Codium fragile* extract as follows: control (0%*_w_*_/*v*_), 100 g of *Codium fragile* extract (at 10 g_dm_ L^−1^ algae concentration) per 1 L of dressing solution (10%*_w_*_/*v*_), 200 g of *Codium fragile* extract (at 10 g_dm_ L^−1^ algae concentration) per 1 L of dressing solution (20%*_w_*_/*v*_), and 300 g of *Codium fragile* extract (at 10 g_dm_ L^−1^ algae concentration) per 1 L of dressing solution (30%*_w_*_/*v*_). These four treatments were applied for each growth condition and respecting the application rate of 1 kg solution per 100 kg seed to be industrially viable. Seed treatment was applied by mixing 1 g of dressing solution with 100 g of seeds and shaking them in a weighing bottle. The choice of these percentages was based on reference values found in the literature [51] and with the aim of discovering a possible dose–response effect and to find the best application dose. Four replicates (each consisting of 100 seeds) were used for each treatment. The treated seeds were placed into a Petri dish (120 mm diameter), with three layers of Whatman Grade 1 filter paper, soaked in the different saline solutions, and left for 4 days in a seed germination cabinet, in dark conditions, at a constant temperature of 20 ± 1 °C [59]. The Petri dishes were randomly allocated within the cabinet. After 4 days, every replicate was transferred into another Petri dish with three new layers of filter paper soaked in the same saline solutions in order to maintain a constant salinity level, and then put back in the germination cabinet at the same temperature for another 4 days, thus obtaining a total germination time of 8 days.

### 2.3. Measured Traits

Germination was determined based on radicle emergence. The germinated seeds of each replicate were counted every day to calculate the following:

- Final germination percentage: (seeds germinated/total seeds) × 100, after 8 days;
- T50: time to achieve 50% of final/maximum germination [60], calculated according to the following formula [61,62]:

(1) $${T50=t}_{i}+\frac{\left(\frac{N}{2}-n_{i})(t_{j}-t_{i}\right)}{\left(n_{j}-n_{i}\right)}$$

where N is the final number of germinated seeds and n_j_ and n_i_ are the cumulative number of seeds germinated by adjacent counts at times t_j_ and t_i_, respectively, when n_i_ < N/2 < n_j_;

- Germination time course.

At the end of the experiment (after 8 days), all germinated seeds were analysed, and the following parameters were measured for each replicate:

- Number of roots, manually counted;
- Length of the main root (cm), measured with a ruler;
- Coleoptile length (cm), measured with a ruler;
- Root biomass (dry weight, g), for which all the roots of each replicate were separated from the germinated seeds and dried off for 2 days at 60 °C;
- Coleoptile biomass (dry weight, g), for which all the coleoptiles of each replicate were separated from the germinated seeds and dried off for 2 days at 60 °C.

### 2.4. Statistical Analysis

Germination data were analysed as time-to-event data, thus allowing a better interpretation of germination experiments [63]. Specifically, relevant parameter estimates (e.g., T50 and final germination) and corresponding standard errors were extracted according to the event-time approach by fitting a three-parameter log-logistic model to data from each germination curve separately [64]. The model equation used in this study was as follows:

(2) $$F(T)=\frac{d}{1+\exp[b\left\{\log\left(T\right)-\log\left(T50\right)\right\}]}$$

where d denotes the proportion of seeds that germinated during the experiment (upper limit or final maximum germination) and T50 is the time (number of days) to reach 50% of final germination. The parameter b is proportional to the slope of F at time T equal to the parameter T50.

Quantitative data obtained from germinated seeds were subjected to analysis of variance (ANOVA) to test the effect of salinity, SWE, and their interaction. Significantly different means were separated at the 95% probability level using Fisher’s protected least significant difference.

Statistical analysis was carried out using the open-source environment R [65] with the add-on packages ‘drcte’, for event-time models [66].

## 3. Results

### 3.1. Germination Percentage and Median Germination Time (T50)

**Figure 1:**
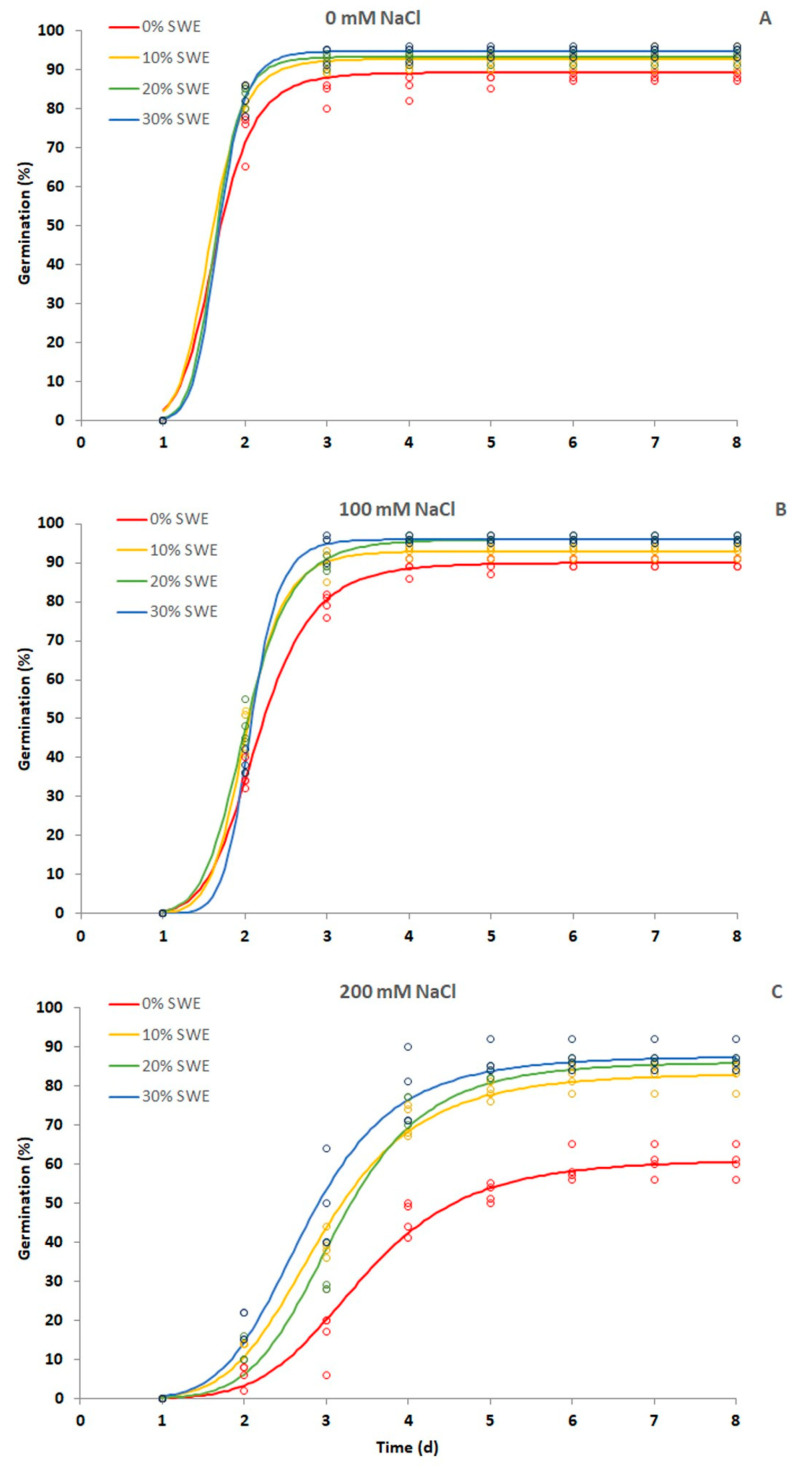
Germination curves generated with the three-parameter log-logistic model of seeds treated with different seaweed extracts (SWE) doses under the following conditions: (**A**) optimal conditions (0 mM NaCl); (**B**) mid-saline conditions (100 mM NaCl); (**C**) high-saline conditions (200 mM NaCl).

As shown in Table 1 and Figure 1, the priming with SWE strongly influenced the germination performance of durum wheat seeds, especially at high salinity. The application of SWE in the control treatment allowed seeds to significantly enhance their final germination. Specifically, final germination increased by 3% at 10% SWE concentration, by 4% at 20% SWE concentration, and by 5% at 30% SWE concentration. At the medium salinity level (100 mM NaCl), the seeds primed at 20% and 30% SWE concentration reached a final germination (96%) significantly higher than that of the control and the treatment with the lowest SWE concentration (90 and 93%, respectively).

The effect of the seed treatment dramatically increased at the highest salinity level (200 mM NaCl). Indeed, under that growth condition, while all the treated seeds showed a final germination percentage ranging from 82% (10% SWE) to 88% (30% SWE), untreated seeds stopped around 61% germination rate.

As for median germination time, expressed as T50, the SWE coating did not significantly affect the seeds’ response in optimal conditions. Conversely, at medium and high salinity levels, the median germination time of the seeds treated with SWE was significantly reduced as compared to that of the control treatment, except for 20% SWE at 200 mM NaCl.

### 3.2. Number of Roots

**Figure 2:**
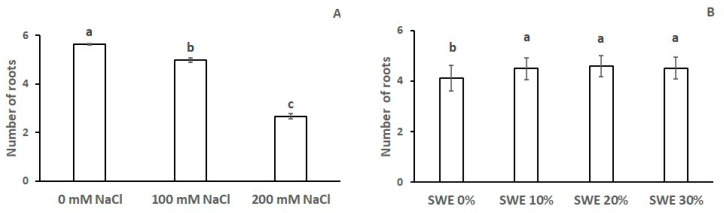
Number of roots as affected by (**A**) salinity and (**B**) seaweed extracts (SWE). The vertical bars represent the standard error of the mean. Letters above the histograms correspond to the ranking of the Fisher’s protected test at *p* < 0.05.

As shown in Table 2, the number of seminal roots was significantly influenced by both SWE and salinity. In more detail, salinity markedly reduced the number of roots, which dropped from 5.6 at optimal conditions to 5.0 and 2.67 at medium and high salt concentrations, respectively (Figure 2A); meanwhile, the application of *Codium fragile* extracts significantly increased the number of roots as compared to the control (up to 12%), irrespective of dose (Figure 2B).

### 3.3. Length of the Main Root

**Figure 3:**
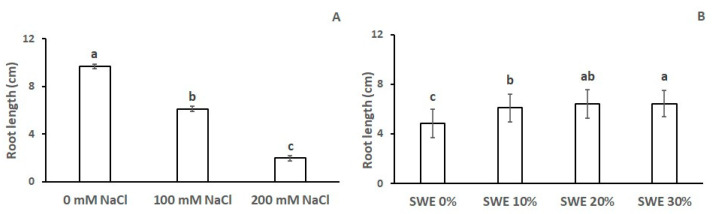
Length of the main root as affected by (**A**) salinity and (**B**) seaweed extracts (SWE). The vertical bars represent the standard error of the mean. Letters above the histograms correspond to the ranking of the Fisher’s protected test at *p* < 0.05.

As with the number of seminal roots, the length of the main root was also significantly affected by salinity and SWE application (Table 2). Salinity reduced the root length by 37% at 100 mM NaCl and by 67% at 200 mM NaCl (Figure 3A). The presence of *Codium fragile* extract in the seed coating markedly increased the root length by 27% at the lowest SWE dose (from 4.8 cm to 6.1 cm) and by 33% at the highest one (from 4.8 cm to 6.4 cm), with a significantly different effect between these two SWE concentrations (Figure 3B).

### 3.4. Root Biomass

**Figure 4:**
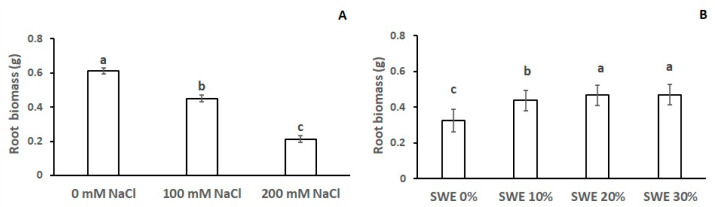
Root biomass, as affected by: (**A**) salinity and (**B**) seaweed extracts (SWE). The vertical bars represent the standard error of the mean. Letters above the histograms correspond to the ranking of the Fisher’s protected test at *p* < 0.05.

As reported in Table 2, root biomass was significantly influenced by both treatments, like the previously reported traits. Specifically, the increasing salt concentration produced a 26% (from 0.61 g to 0.45 g) and 66% (from 0.61 g to 0.21 g) reduction of root dry weight (Figure 4A). The application of SWE increased root biomass by 35% and 42% at the lowest and highest concentrations, respectively (Figure 4B). No significant difference was detected applying SWE at 20% or 30% concentration.

### 3.5. Coleoptile Length

**Figure 5:**
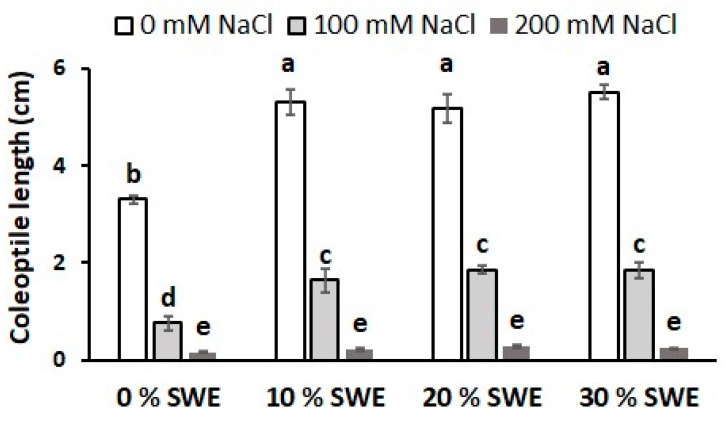
Length of the coleoptile as affected by salinity × SWE interaction. The vertical bars represent the standard error of the mean. Letters above the histograms correspond to the ranking of the Fisher’s protected test at *p* < 0.05.

The interaction between treatments was found to be significant for this trait (Table 2). As shown in Figure 5, the application of *Codium fragile* extract was greatly effective for the control treatment (+60% in coleoptile length) and at medium salt stress conditions (coleoptile length doubled), while no remarkable benefit was detected under high salt stress conditions. As previously observed for the other traits, the increase in salt stress severely hindered the development of wheat coleoptile.

### 3.6. Coleoptile Biomass

**Figure 6:**
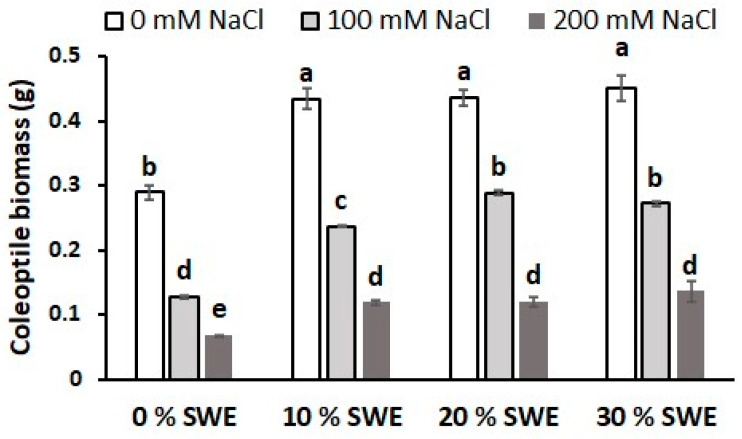
Coleoptile biomass as affected by salinity × SWE interaction. The vertical bars represent the standard error of the mean. Letters above the histograms correspond to the ranking of the Fisher’s protected test at *p* < 0.05.

The interaction between treatments significantly affected coleoptile total biomass (Table 2). The increase in salt concentration strongly reduced the biomass, moving from an average of 0.4 g for the optimal conditions to 0.23 g for 100 mM NaCl and 0.11 g for 200 mM NaCl (Figure 6). The application of SWE increased the performance in all growth conditions. In optimal conditions, all the biostimulant-coated seeds had a higher biomass compared to the control, with a 52% increase. At the medium salinity level, the application of 20% and 30% SWE enhanced the biomass weight compared with the 10% SWE. However, all the SWE-treated seeds performed significantly better than the control. At the high salinity level (200 mM NaCl), all the seed coating treatments enhanced the coleoptile’s biomass by 79% as compared to the control.

## 4. Discussion

According to our first hypothesis, the *Codium fragile* extract significantly enhanced seed germination and seedlings development in each growing condition, especially under salt stress. Conversely, the second hypothesis was not verified since, in most of the measured traits, there is not a clear dose–response effect for the application of *Codium fragile* extract. This means that the SWE is effective also at lower doses.

Furthermore, the results showed that the seed coating can be a viable way to deliver SWE and so it could be applied at an industrial scale too.

According to our findings, seed germination in durum wheat appeared to be stable until medium salt stress (100 mM NaCl), while it was reduced by 30% under high salt stress conditions (200 mM NaCl). This result agrees with other studies found in the literature [67,68,69,70]. Even though final germination at 100 mM NaCl did not differ from that of optimal conditions, seeds evidenced a longer median germination time (T50) and a lower coleoptile length. That negative effect will certainly lead to a plant being more susceptible to biotic and abiotic stresses, thus reducing yield and grain quality [22,71,72,73]. A fast and uniform seed germination, combined with a high seedling vigour, are the fundamental traits used to assess salt tolerance in crops [39,73,74].

Generally, the application of SWE significantly enhanced all the measured traits, except for the coleoptile length under high salt stress. Even though literature on this specific research topic is limited, the positive effect of SWE, and especially of *Codium* spp. extract, in promoting wheat germination (also in controlled salinity conditions) are reported in other studies [51,52]. *Codium* spp., as other macroalgae, appear to be rich in phytohormones such as cytokinin, gibberellins, and auxins [75,76,77]. These bioactive compounds are certainly involved in the germination process, and their presence can stimulate the metabolic process in wheat seeds [78,79,80]. Auxins seem to play a major role in promoting seed germination and seedlings development under salt stress conditions. Even though these plant growth regulators are fundamental for the early development of the plants, the stored auxins are not sufficient to guarantee a good stem and roots formation, especially under salt stress [79]. Indeed, plants rapidly decrease the production of auxins during salt stress, triggering down the auxin biosynthesis pathways [81]. Thus, the application of endogenous auxins could fulfil the plants’ requirements for wheat germination and seedlings development [79,82]. In addition to that, these phytohormones appear to promote tissue regeneration and limit the uptake and assimilation of toxic mineral elements [79,83]. The cytokinins are also involved in the salt stress regulation, especially in the early stage of the seedling’s development [84]. It has been reported that salinity significantly reduces the cytokinins’ export from the root to the shoot [85]; therefore, an exogenous application of cytokinins could lead to a better development of the wheat seedlings under salt stress conditions [80]. Additionally, the SWE tested contains amino acids such as proline and glycine betaine [51,52,86]. These two amino acids are well known to enhance crop resistance to drought and salt stress [87,88,89]. Glycine betaine seems to reduce toxic ions’ absorption, protecting ion channels and favourably influencing the properties of the cell membrane. This mechanism greatly favours ion homeostasis under salinity stress [90]. As for proline, this amino acid has proven to limit the negative impact of the salt stress thanks to its role as an osmoprotectant, membrane stabilizer, and scavenger of reactive oxygen species [91,92]. The strong effect of the *Codium fragile* extract is probably due to the combination of these different active compounds that, through different modes of action, confer to durum wheat seedlings a greater tolerance to salt stress. Other studies reported that priming seeds with SWE leads to an improved antioxidant enzyme activity [42]. The seed coating with SWE has also led to a general reduction in germination time, especially under salt stress conditions. Again, this effect could be explained by the presence of phytohormones in the *Codium fragile* extract, as these compounds are able to promote the creation of hydrolytic enzymes and are inhibitors of abscisic acid [93].

As expected, the seedlings’ vigour was negatively affected by salinity. In particular, the coleoptile and the root system showed a strong reduction in terms of both length and total dry biomass, even under medium salt stress. This result is consistent with other studies conducted on winter cereals, reporting a different reduction of root length (from 20% to 97%) and dry biomass (from 60% to 70%), depending on the salinity level and the different growing conditions. A similar effect has been shown in shoot length, with reduction rates ranging from 36% to 92% [69,94,95,96]. However, the application of *Codium fragile* extract, especially at the highest doses, was able to alleviate the salt stress, as evidenced by the improved length and biomass of the coleoptile in SWE-treated seeds. Compared to the control treatment, the application of SWE markedly promoted the development of the root system. A similar positive effect on shoots and roots was observed also in other crops such as tomato, okra, etc. [36,42,97]. It is well known that biostimulants, especially seaweed extracts, can increase crop development, even in stress conditions [33]. This effect is likely due to the presence of phytohormones, as well as the content of macro- and micronutrients. In particular, in *Codium* species, the effect can be due to the presence of elements such as calcium and zinc [86,98]. The exogenous application of these two elements resulted in a general enhancement of the germination percentage and seedling vigour [99,100,101].

## 5. Conclusions and Future Prospects

Seed treatment with biostimulants is still a relatively under-explored research topic, but it appears extremely interesting. Our study clearly demonstrated that seed coating with a *Codium fragile* extract was effective in enhancing seed germination and early vigour of the durum wheat cultivar Monastir, both under optimal (0 mM NaCl) and salt stress conditions (100 and 200 mM NaCl). A prompt and uniform seedling emergence, combined with a better rooting, can make crop species more tolerant to biotic and abiotic stresses. Moreover, the application of seaweed extracts could be a promising way to decrease the use of synthetic fertilizers in agriculture, without negatively affecting yield potential. The future investigation into all these aspects can greatly improve our understanding of sustainable management of durum wheat. Additional studies will be necessary to gain a deeper understanding of the mechanism of action of this biostimulant. Moreover, since we reported results from a germination trial only, several field experiments will be needed to evaluate the effects on plants during the entire growing cycle, under different soils and climatic conditions. In that way, we will also be able to explore other *Codium fragile* application methods, such as foliar spraying or soil treatments.

## Figures and Tables

**Table 1:** Final germination percentage and median germination time (T50) for each combination of growth condition and SWE treatment ± standard errors.

Growth Condition	SWE	Final Germination (%)	T50 (d)
Salinity 0 mM NaCl	0%	89.34 ± 1.28 b	1.65 ± 0.02 a
	10%	92.70 ± 1.45 a	1.62 ± 0.05 a
	20%	93.13 ± 0.96 a	1.71 ± 0.05 a
	30%	94.82 ± 0.79 a	1.73 ± 0.03 a
Salinity 100 mM NaCl	0%	90.10 ± 0.55 c	2.15 ± 0.01 a
	10%	92.79 ± 0.77 b	2.03 ± 0.04 b
	20%	96.05 ± 0.60 a	2.01 ± 0.03 b
	30%	96.05 ± 0.60 a	2.04 ± 0.01 b
Salinity 200 mM NaCl	0%	60.67 ± 1.08 c	3.43 ± 0.11 a
	10%	82.21 ± 1.22 b	2.95 ± 0.07 bc
	20%	85.65 ± 0.51 ab	3.13 ± 0.07 ab
	30%	87.70 ± 1.54 a	2.77 ± 0.12 c

Within each growth condition, means sharing letters are not significantly different at *p* < 0.05.

**Table 2:** Results of the ANOVA for quantitative traits of germinated seeds.

	Number of Roots	Length of the Main Root	Root Biomass	Coleoptile Length	Coleoptile Biomass
SWE	***	***	***	***	***
Salinity	***	***	***	***	***
SWE × Salinity	n.s.	n.s.	n.s.	***	***

ANOVA signif. codes: ‘***’ < 0.001; n.s.: not significant.

## Data Availability

The data presented in this study are available on request from the corresponding author.

## Abbreviations

SWE	seaweed extract
0% SWE	seed dressing solution containing 0 g of *Codium fragile* extract
10% SWE	seed dressing solution containing 100 g of *Codium fragile* extract
20% SWE	seed dressing solution containing 200 g of *Codium fragile* extract
30% SWE	seed dressing solution containing 300 g of *Codium fragile* extract
T50	median germination time
